# Ventrofixation of Uterus

**Published:** 1907-01

**Authors:** 


### VENTROFIXATION OF UTERUS.

The operation, according to Giles, is of value:

1. In cases of movable retroversion of the uterus when pessaries have been tried without success; when the uterus retains its position as long as the pessaries are worn, but falls back as soon as they are removed; and in cases in which the patient prefers a radical procedure to pessary treatment and the consequent repeated examinations. 2. In cases in which the uterus is held down by adhesions in a position of retroversion. 3. In cases in which a retroversion is complicated by disease of the appendages. 4. In cases of prolapse and of total procidentia.

Coexistent abnormal conditions, he says, should be treated at the same time whenever possible. Thus, when there has been menorrhagia or endometritis the uterus should be cureted; a torn cervix should be repaired; a hyperplastic cervix should be amputated; small and solitary fibroids should be removed; prolapse of the vaginal walls should be treated by colporrhaphy or perineorrhaphy, or both; and diseased appendages should be treated by conservative procedures, or by removal, according to the nature of the case.

As to the results of ventrofixation, Giles claims that in the event of pregnancy following operation there is a slightly increased risk of abortion, but about 80 per cent. of patients have an undisturbed pregnancy and a normal confinement, and the operation does not entail any added risk at the time of labor. Pregnancy has a slight disturbing influence on the position of the uterus, but in over 80 per cent. of cases the uterus maintains its position, in spite of pregnancy. In cases not followed by pregnancy the operation is permanently successful in 95 per cent. The adominal scar gives trouble in only 2 or 3 per cent. of cases. The operation leads to marked improvement in the patient's general condition in about 90 per cent. of cases.—*Brittish Medical Journal,* November 3, 1906.

